# Making progress in non-human mental time travel

**DOI:** 10.3389/fpsyg.2014.00305

**Published:** 2014-04-09

**Authors:** Corina J. Logan

**Affiliations:** Department of Psychological and Brain Sciences, SAGE Center for the Study of the Mind, University of California Santa BarbaraSanta Barbara, CA, USA

**Keywords:** mental time travel, episodic memory, future planning, non-human, imagination

Humans can remember unique past events and plan for the future and they can imagine themselves at these events when they are not currently occurring, an ability often called mental time travel and thought to be distinctly human (Suddendorf and Corballis, 2007). The behavior of many non-human species indicates that they can also remember unique past events to plan for the future, however it is not known whether they actually imagine themselves outside of the present (Clayton et al., 2003).

Corballis (2013a,b) recently commented on new research on the hippocampus (a brain region involved in spatial navigation) showing that rats may be able to imagine themselves in situations other than their current one because when resting they replay particular neurological sequences indicated by the firing of “place cells” associated with familiar and novel spatial trajectories (Gupta et al., 2010). Corballis considers this possible evidence that non-humans imagine themselves in the past and future and, thus, mentally travel in time. I agree that non-humans likely do imagine themselves in the past and future, however, it is important to note that while both humans and non-humans activate these neurological sequences to engage in goal-directed behavior (Ekstrom et al., 2003), there is no direct evidence yet that these sequences indicate that imagination or planning is involved (Gupta et al., 2012). These studies lack evidence from brain imaging studies that link imagination and planning to the neurological sequences associated with spatial trajectories.

Brain imaging studies would also help evaluate Corballis (2013a,b) and Suddendorf's (2013) claim that human mental time travel is more complex than that in non-humans because we can imagine not only locations, but also other aspects of particular scenarios including “… people, things, places, [and] actions” (Corballis, 2013a, p. 5). It is too early to arrive at this conclusion due to the lack of research investigating imagination in non-humans as well as the absence of non-human mental time travel experiments that examine behavior and neurological activity at the same time. To understand what mental processes are involved in mental time travel, we must look at what mental processes are occurring in the brain when performing behavioral experiments. Investigating the question of what non-humans can imagine requires studying neurological activity across the whole brain, not just the hippocampus, since brain areas outside of the hippocampus are active when humans imagine other individuals, objects, and actions (e.g., Decety, 1996; Hassabis et al., 2013; Schlegel et al., 2013; see Polyn and Sederberg, 2014 for a review). Investigations of whole brain activity combined with creative experimental designs could determine whether non-humans use imagination to mentally travel in time.

## Examining whole brain activity

While the advancement of technology will open avenues for studying neurobiology at greater spatial and temporal resolutions, progress is also being made using existing technology in new ways (e.g., examining bird brain activity from real-time behavior using micro-PET scans: Marzluff et al., 2012; Cross et al., 2013). These advances have broadened our ability to test hypotheses about the complexity of non-human cognition.

Investigations of whether imagination and planning are involved in the replay of neurological sequences associated with space will benefit from a research approach that combines technologies. One such approach could use the hippocampal tetrodes that record place cell activity in conjunction with electrocorticography (ECoG) and positron emission tomography (PET) to detect which other brain regions are active during replay events. ECoG has a high temporal resolution (on the order of milliseconds) and the implanted electrodes allow the animal to behave normally (i.e., not anesthetized in a scanner; Buzsáki et al., 2012). PET has a low temporal resolution, but allows a higher resolution of active brain areas thus complimenting the lower spatial resolution of ECoG. PET also allows brain activity to be examined in the context of normal behavior because the scans detect positively charged particles that result from the metabolism of a radioactive glucose tracer. It takes several minutes for the tracer to be metabolized sufficiently to represent all of the neural activity during the time of interest, thus giving experimenters time to conduct the behavioral trials and then anesthetize and scan the subject. Indeed, a study using ECoG and PET scans found that results from the two methods were significantly in agreement and together they provided a higher resolution than each method could when used in isolation (Chandra et al., in press). Used together during mental time travel experiments, ECoG, PET, and hippocampal tetrodes could begin to illuminate whether brain areas involved in imagination and planning are active during replay events and, thus, whether non-humans imagine themselves in the past and future.

Identifying which areas in the brains of non-humans are involved in imagination and planning remains a challenge since brain structures vary across species, thus inferences cannot necessarily be based on human brain activity and anatomy (e.g., Krubitzer et al., 2011). Yet this lack of knowledge gives even more strength to the argument to examine activity from the whole brain to facilitate a solution to this problem.

## Creative experimental designs

Experiments that require the subject to replay neurological sequences associated with spatial trajectories while at rest will be useful because they should activate the brain areas involved in imagination and planning. For example, using the Gupta et al. (2010, 2012) spatial maze, each of the four feeders could have a unique color and contain food that is more or less preferred, thus establishing an order of preference for the feeders. Keeping the subject at the starting position in the maze, a photo or video of the feeder with the preferred food shown to the resting animal should evoke the neurological sequences associated with the spatial trajectory for traveling to that feeder (see Miller et al., 2013 for a similar experimental design used for humans). Using this same paradigm, experimenters could incorporate a time component by having the preferred feeders only dispense food after a delay, while the least preferred food is always available. If the preferred foods are not available when the image is shown because an insufficient amount of time has passed for them to become accessible again, then the subject should choose the least preferred and always available feeder as represented by the neurological sequence it replays. These experimental conditions should be contrasted with control conditions in which the only difference is that no imagination is required. For instance, showing the animal a novel picture (e.g., a white background). This would test whether the animal remembers *what* food is *where* and *when* it should run to a particular feeder. What, where, and when are the three components of mental time travel that are detectable by observing behavior (Clayton et al., 2003). This paradigm uses an experimental design similar to that used for western scrub-jays (Clayton et al., 2003) and adds the neurobiological component necessary to determine whether non-humans also engage imagination.

## Conclusion

To answer the question of whether non-humans imagine themselves in the past and future it is necessary to go beyond behavioral studies and investigate behavior in conjunction with brain activity. By examining activity across the whole brain in the context of creative experimental designs that test conditions requiring imagination against controls that do not, it should be possible to determine whether they imagine themselves not only in particular places, but also in the context of other individuals, objects, and actions. It is wise to reserve judgment on the distinctness of humans until comparable data, especially on the brain activity behind the behavior, exist in non-human species.

### Conflict of interest statement

The author declares that the research was conducted in the absence of any commercial or financial relationships that could be construed as a potential conflict of interest.
